# Retraction Note: circular RNA circ-CSPP1 regulates CCNE2 to facilitate hepatocellular carcinoma cell growth via sponging miR-577

**DOI:** 10.1186/s12935-023-02989-5

**Published:** 2023-07-17

**Authors:** Qian Sun, Rui Yu, Chunfeng Wang, Jianning Yao, Lianfeng Zhang

**Affiliations:** Department of Gastroenterology and Hepatology, The First Affiliated Hospital of Zhengzhou University, 1 Jianshe Dong Lu, Erqi District, Zhengzhou, 450052 Henan China

**Cancer Cell International (2020) 20:202**

10.1186/s12935-020-01287-8.

The Editors-in-Chief have retracted this article. Concerns were raised regarding Fig. 3g, which appears to overlap with Fig. 2h in an article by different authors that was simultaneously under consideration with another journal [1].

The Editors-in-Chief therefore no longer have confidence in the results and conclusions of this article. The authors have not responded to correspondence regarding this retraction.
